# Supplementary material for: First record of the smallest extant brachiopod Gwynia capsula (Jeffreys, 1859) from the German Bight
Source: PLoS One. 2026 Jan 27;21(1):e0341600. doi: 10.1371/journal.pone.0341600 (PMC12843560; doi:10.1371/journal.pone.0341600)
Supplement: Attachment — Submitted filename: Response to Reviewers.pdf [file pone.0341600.s001.pdf]

MUSEUM FÜR NATURKUNDE BERLIN · INVALIDENSTRASSE 43 · 10115 BERLIN

To
Mikolaj K. Zapalski
Academic Editor
PLOS One

MUSEUM FÜR NATURKUNDE
BERLIN

Leibniz-Institut für Evolutions- und Biodiversitätsforschung

Prof. Dr. Carsten Lüter
Kustos „Marine Wirbellose“ und "Vermes" (comm.)

Tel +49 30 889140 8529
Fax +49 30 889140 8565
Mail carsten.lueter@mfn.berlin

www.museumfuernaturkunde.berlin

Response to Reviewers, PONE-D-25-60587

Berlin, 06.01.2026

Dear Prof. Zapalski, dear reviewers,
thank you for giving us the chance to revise our manuscript according to your comments and recommendations. As requested, we will give a full list of changes made to the manuscript in the following.

In general, we have changed the layout of the manuscript (title page, headings, reference style etc.) to meet the requirements given in the formatting samples.

Regarding the full ethics statement requested under #2 of your email, I have now added an ethics statement in Material and methods, which hopefully meets the standards of PlosONE. As research was conducted in Germany, only vertebrates and cephalopods fall under the German Animal Welfare Act (Tierschutzgesetz), therefore no ethical approval was required for this study.

In your email, you mentioned that both figures 1 and 2 contain copyrighted material, which needs either being granted for publication under the CC BY 4.0 license by the copyright holder(s) or deleted altogether. Regarding Fig. 1, you were right, since I used a published map of Helgoland to re-draw the map in Fig. 1A. This has now been replaced by a map re-drawn from an open source (OpenStreetMap) which meets the respective requirements. As given in the amended manuscript text, the figure caption refers to OpenStreetMap as a source and needs to be linked to openstreetmap.org/copyright. However, Fig. 2 exclusively contains illustrations and images produced by us, so there is no foreign copyright involved. To support this, I amended the M&M section including data on µCT-methods as well as the acknowledgments to thank our inhouse µCT-technician for her help.

As suggested in the formatting guidelines, I inserted the figure captions into the text following the paragraph in which the respective figure is first mentioned.

@Academic Editor Mikolaj Zapalski: The inventory number of the *G. capsula* specimen from Helgoland, ZMB Bra 2496, has been added to the M&M

Prof. Johannes Vogel, Ph.D.
Generaldirektor

Stephan Junker
Geschäftsführer

Deutsche Bank AG
IBAN: DE40 1007 0848 0512 0878 00
BIC/SWIFT: DEUTDEDB110

Leibniz Gemeinschaft

section. The Krause brothers visited the Tlingit in Alaska, now added in the text. In their German publication they named them "Tlinkit", but all online resources now give the tribe's name as "Tlingit", probably following the indigenous pronunciation; I have amended this in the text accordingly.

@Reviewer #1: Thanks for your positive review and your recommendation to publish the manuscript as is. Much appreciated.

@Reviewer #2: The publication of Metzger (1875) has been added to the reference list. The citation of Caspers has been corrected to 1939 in the text. The reference of Hansen (2024) has been added to the citations and led to studying the original description of the mentioned Holocene fossil by Sars (1865), also added to references. As this comment ties in to what reviewer 3 also mentioned, namely the possibility that *G. capsula* may be present in the region for a much longer time than suggested, I added some text in the "Life style" sub-chapter of the Discussion and a sentence at the end of its last sub-chapter to integrate both the information from the Sars and Hansen papers and to add natural dispersal over more than a million years as an alternative explanation for the recent findings of the brachiopod on Helgoland.

@Reviewer #3 (see also above): Natural migration over longer periods of time is indeed a possibility. The fact that *Gwynia*'s minute size has certainly led to a sample bias since they are easy to miss in sediment samples was already mentioned by us in the original manuscript. However, this opens the possibility of a much wider distribution of the species in the North Sea than previously thought, so does the finding of the Holocene fossil by Sars in 1865, a paper which was indeed not known to us. In both, Sars (1865) and Hansen (2024) the single fossil specimen (which unfortunately seems to be lost!) was said to be of Holocene, not of Pleistocene origin. We have written two short paragraphs in the discussion, mentioning both papers and the possibility of a natural dispersal over the last million or so years.

I hope that our corrected manuscript now meets the requirements of the journal. I am looking forward to receiving your final recommendation.
On behalf of all authors with best wishes

Prof. Dr. Carsten Lüter
